# Comparison of endoscopic thyroidectomy by complete areola approach and conventional open surgery in the treatment of differentiated thyroid carcinoma: A retrospective study and meta-analysis

**DOI:** 10.3389/fsurg.2022.1000011

**Published:** 2022-12-20

**Authors:** Yuquan Yuan, Chenyu Sun, Tingjie Yin, Cong Shao, Bin Pan, Dengwei Lu, Shaodong Hou, Scott Lowe, Rachel Bentley, Shuya Chen, Christy Huang, Ce Cheng, Yaru Li, Bethany King, Qin Zhou, Cunye Yan, Fan Zhang

**Affiliations:** ^1^ Department of Breast and Thyroid Surgery, Chongqing General Hospital, Chongqing, China; ^2^ Graduate School of Medicine, Chongqing Medical University, Chongqing, China; ^3^ Department of Medicine, AMITA Health Saint Joseph Hospital Chicago, Chicago, IL, United States; ^4^ Graduate School of Medicine, North Sichuan Medical College, Nanchong, China; ^5^ College of Osteopathic Medicine, Kansas City University, Kansas, MO, United States; ^6^ Foundation Program, Newham University Hospital, London, England, United Kingdom; ^7^ California Health Sciences University College of Osteopathic Medicine, Clovis, CA, United States; ^8^ Hematology and Medical Oncology, St. Joseph Mercy Ann Arbor Hospital, Ypsilanti, MI, United States; ^9^ Internal Medicine, Swedish Hospital, Chicago, IL, United States; ^10^ Internal Medicine, Mercy One Des Moines Medical Center, Des Moines, IA, United States; ^11^ Radiation Oncology, Mayo Clinic, Rochester, MN, United States

**Keywords:** endoscopic thyroidectomy, conventional open thyroidectomy, differentiated thyroid carcinoma, feasibility, meta-analysis

## Abstract

**Background:**

The feasibility of endoscopic thyroidectomy by complete areola approach (ETCA) remains controversial. This study was conducted by combining our clinical data with the data obtained from a systematic review literature search to examine the effectiveness and safety of ETCA compared with conventional open thyroidectomy (COT) in differentiated thyroid carcinoma (DTC).

**Methods:**

A total of 136 patients with a diagnosis of DTC who underwent unilateral thyroidectomy with central neck dissection from August 2020 to June 2021 were enrolled. The enrolled patients were divided into the ETCA group (*n* = 73) and the COT group (*n* = 63). The operative time, intraoperative bleeding volume, number of removed lymph nodes, number of metastatic lymph nodes, postoperative drainage volume, length of postoperative hospital stay, postoperative parathyroid hormone (PTH) levels, and complications were analyzed. Then, a systemic review and comprehensive literature search were conducted by using PubMed, Google Scholar, Embase, Web of Science, CNKI, Wanfang, and VIP database up to June 2022. Review Manager software version 5.3 was used for the meta-analysis.

**Results:**

The results of clinical data showed that there were significant differences between the two groups in the operative time, intraoperative bleeding volume, removed lymph nodes, and postoperative drainage volume. There were no statistical differences in the length of postoperative hospital stay, number of metastatic lymph nodes, postoperative PTH level, and complications. In the systematic review and meta-analysis, 2,153 patients from fourteen studies (including our data) were ultimately included. The results of the meta-analysis found that ETCA had a longer operative time, larger postoperative drainage volume, and lower intraoperative bleeding volume. In terms of the length of postoperative hospital stay, the number of removed lymph nodes, and surgical complications, there was no significant difference between the two groups.

**Conclusion:**

ETCA poses lower surgical bleeding and better cosmetic appearance compared with COT, while the length of operation and postoperative drainage in ETCA is less favorable compared with COT. In addition, ETCA is not inferior to COT in terms of the postoperative hospitalization stay, the number of removed lymph nodes, and surgical complications. Given its overall advantages and risks, ETCA is an effective and safe alternative for patients with cosmetic concerns.

## Introduction

Thyroid cancer, the most common malignancy of the endocrine system, has been multiplying in recent years, ranking 9th for incidence in 2020 (1). Among different types of thyroid cancer, differentiated thyroid carcinoma (DTC), including papillary thyroid carcinoma (PTC) and follicular thyroid carcinoma, accounts for most cases worldwide (2). Conventional open thyroidectomy (COT) is a long-proven and effective treatment for DTC with good outcomes, but it also leaves a prominent scar on the anterior neck. The COT involves creating a 5-cm incision in the skin fold at 2 cm above the sternal notch, and the subcutaneous tissues are then separated layer by layer to fully expose the thyroid gland. With a favorable prognosis (3), many patients consider that maintaining a high quality of life with an aesthetic appearance is as crucial as disease management itself. The cosmetic implications of neck surgery have motivated surgeons and investigators to develop new approaches to accessing the neck using minimally invasive techniques. Since Hüscher et al. first performed endoscopic thyroidectomy (ET) in 1997, an increasing number of studies have reported different ET approaches for treating DTC (4–6). In the procedure of endoscopic thyroidectomy by complete areola approach (ETCA), a 12-mm incision and a 5-mm incision are made in the right mammary areola, while a 5-mm incision is made in the left mammary areola to imbed the puncture sheath, a 30° endoscope, and the operating apparatus. As a result of tiny incisions and less skin tension in the areola region, ETCA allows for the removal of bilateral lesions with excellent cosmetic outcome (7). However, certain indicators that may be used to assess the efficacy and safety between ETCA and COT, such as operative time, number of lymph nodes excised, postoperative parathyroid hormone levels, and incidence of surgical complications remains debatable (8, 9).

Several previous meta-analysis comparing results between ET and COT has been published (10, 11). However, to the best of our knowledge, no meta-analysis investigating ETCA vs. COT has been published. With the increased popularity of ETCA, the number of original studies exploring its safety and treatment outcomes has gradually increased. In this study, along with data retrieved from previously published studies that were searched with the systemic review method, we specifically included the data from one regional academic medical center to perform a meta-analysis to compare the effectiveness and safety of ETCA with COT in DTC patients.

## Materials and methods

### Date collecting

The retrospective study group comprised 136 patients who underwent unilateral lobectomy and central lymph nodes dissection at Chongqing General Hospital from August 2020 to June 2021. The patients were divided into two surgical method groups: the ETCA group (*n* = 73) and the COT group (*n* = 63). All patients had a pathological diagnosis of DTC with tumor sizes ≤ 2 cm. Additionally, all patients with suspicious invasion of the recurrent laryngeal nerve (RLN), esophagus, trachea, suspicious lateral lymph node metastasis, or a history of neck surgery were excluded from the study. All operations were performed by one experienced board-certified surgeon. This study was approved by The Ethical Committee of Chongqing General Hospital, and all patients included signed the informed consent.

Clinical data were collected from medical records, and a database was set up to record patient age, tumor size, operative time, intraoperative bleeding volume, number of removed lymph nodes, number of metastatic lymph nodes, postoperative drainage volume, length of postoperative hospital stay, postoperative parathyroid hormone (PTH) levels and complications (transient hoarseness and postoperative infection). Specifically, operative time, postoperative drainage volume, length of postoperative hospital stay, the number of removed lymph nodes, and the number of metastatic central lymph node were compared between these two operative approaches to assess the surgical effectiveness. The intraoperative bleeding volume, postoperative PTH levels, and the incidence of surgical complications were used to evaluate surgical safety.

### Statistical analysis

SPSS 25.0 software (IBM, Armonk, NY, USA) was used for statistical analysis. Measurement data were expressed as mean ± standard deviation, and t-test was performed for the intergroup comparison. Enumeration data were expressed as rate (%), and *χ*^2^ test was performed for the intergroup comparison. *P* < 0.05 suggested statistically significant difference.

### Meta-analysis

This meta-analysis was reported in conformity to the Preferred Reporting Items for Systematic Reviews and Meta-Analyses (PRISMA) (12). In addition, the study protocol was prospectively registered in PROSPERO (http://www.crd.york.ac.uk/PROSPERO; registration number: CRD42022344008).

### Literature search strategy

The articles up to June 2022 were collected from PubMed, Google scholar, Embase, Web of Science, CNKI, Wanfang and VIP database. The keywords were (“Areola” OR “complete areolar” OR “endoscopic thyroidectomy”) AND (“thyroid” OR “thyroid cancer” OR “thyroid carcinoma”). In addition, reference lists of the retrieved articles were reviewed to identify other eligible studies.

### Identification of eligible studies

Two authors (Y. Yuan and T. Yin) independently carried out the literature search and disagreements were solved by consensus. The abstracts of the retrieved studies were reviewed and excluded if deemed irrelevant. The full text of the relevant studies was further reviewed for eligibility. If there were duplicate publications of the same study, the one with the most detailed information and complete data was included.

Studies included in this meta-analysis must meet al.l of the following criteria: (1) The type of study must be either a randomized controlled trial or an observational study (including cohort and case-control studies). (2) Have complete or computationally extractable data. (3) The experimental group must undergo ETCA. (4) The studies were published in English or Chinese. The exclusion criteria were set for this study. (1) The type of articles cannot be accurately determined. (2) No valid account of ending data can be derived from the article. (3) Duplicate articles. (4) Review, animal studies, case reports, etc.

### Data extraction and quality evaluation

Each of the two authors (Y. Yuan and T. Yin) independently disposed the data from the literature, and if discrepancies arose, a consensus was reached by consulting a third person (C. Yan) and comprehensively comparing the data. Information was collected as follows: first author, country, publication year, age, tumor size, operative time, intraoperative bleeding volume, number of removed lymph nodes, postoperative drainage volume, length of postoperative hospital stay, hoarseness, hypocalcemia, hematoma, infection, study design, and Newcastle–Ottawa Scale (NOS) scores.

Methodological quality of the observational researches was appraised using a validated NOS. Three broad subscales including study group selection (0 to 4 points), the groups comparability (0 to 2 points), and the exposures and outcomes elucidation (0 to 3 points). A score of 4–6 was considered moderate, and a score of 7 or more is defined as high quality. Two evaluators (C. Yan and Y. Chen) independently conducted the quality assessment and if there was a divergence of opinion, it would be resolved by mutual communication.

### Statistical analysis and publication bias evaluation

RevMan software (version 5.3; Cochrane Library) and STATA statistical Software (version 14.0; StataCorp, College Station, TX) were used for statistical analysis. Continuous data were analyzed using weighted mean difference (WMD) with corresponding 95% confidence interval (CI), whereas dichotomous data were measured using odds ratio (OR) with corresponding 95% CI. Heterogeneity tests were performed based on Q test and *I^2^* statistics. For *I^2^ *> 50%, the random effects model was implemented to create forest plots. In contrast, the fixed-effects model was adopted if *I^2^* was <50%. Sensitivity analysis was performed by sequentially excluding each incorporated study at one time and observing whether the combined results changed significantly (13). Publication bias was assessable on a funnel plot qualitatively (14). All *P*-values were two-tailed and *P* < 0.05 was considered statistically significant.

## Results

### Comparison between the two groups

All patients in both groups underwent unilateral thyroidectomy and central lymph nodes dissection. The operative time in ETCA group were significant longer than that in COT group (114.72 ± 25.62 vs. 102.96 ± 22.79, *P *= 0.006). The intraoperative bleeding volume in the ETCA group, was significantly less than that in COT group (18.38 ± 7.77 vs. 35.67 ± 11.42, *P *< 0.001). The number of removed lymph nodes in the ETCA group was more than that in the COT group (8.84 ± 6.52 vs. 6.41 ± 5.47, *P *= 0.022) and the number of metastatic lymph nodes was similar between two groups (0.59 ± 1.24 vs. 0.59 ± 1.61, *P *= 0.994). The postoperative drainage volumes in the ETCA group were more than that in the COT group (195.75 ± 80.10 vs. 137.83 ± 52.09, *P *< 0.001). There was no significant difference of the length of postoperative hospital stay between the ETCA and COT groups (3.68 ± 0.88 vs. 3.59 ± 1.10, *P *= 0.567). In the term of postoperative PTH levels, there was no significant difference between the ETCA and COT groups (34.59 ± 12.18 vs. 36.86 ± 11.43, *P *= 0.268). In regards to incidence of postoperative complications, there were no significant differences between the two groups. For instance, only 2.7% and 3.1% patients developed short-term hoarseness that recovered within two weeks in the ETCA group (2/73) and COT group (2/63), respectively. Postoperative infection is a rare complication that only occurred in 1.3% and 1.6% patients in the ETCA group (1/73) and COT group (1/63), respectively (Table 1). No postoperative bleeding hypocalcemia, unanticipated secondary surgery or other serious complications had occurred in any of the patients in the two groups.

**Table 1:** Comparison of indicators between the two groups.

Indicators	ETCA group (*n* = 73)	COT group (*n* = 63)	*P* value
Age (year)	32.51 ± 6.03	36.00 ± 8.01	0.005
Tumor size (cm)	0.64 ± 0.77	0.57 ± 0.24	0.497
Operative time (min)	114.72 ± 25.62	102.96 ± 22.79	0.006
Intraoperative bleeding volume (ml)	18.38 ± 7.77	35.67 ± 11.42	<0.001
Number of removed lymph nodes	8.84 ± 6.52	6.41 ± 5.47	0.022
Number of metastatic lymph nodes	0.59 ± 1.24	0.59 ± 1.61	0.994
Postoperative drainage volume (ml)	195.75 ± 80.10	137.83 ± 52.09	<0.001
Length of postoperative hospital stay (days)	3.68 ± 0.88	3.59 ± 1.10	0.567
Postoperative PTH levels (pg/ml)	34.59 ± 12.18	36.86 ± 11.43	0.268
Transient hoarseness	2	2	0.882
Postoperative infection	1	1	0.917

ETCA, endoscopic thyroidectomy by complete areola approach; COT, conventional open thyroidectomy.

## Meta-analysis

### Study selection

**Figure 1:**
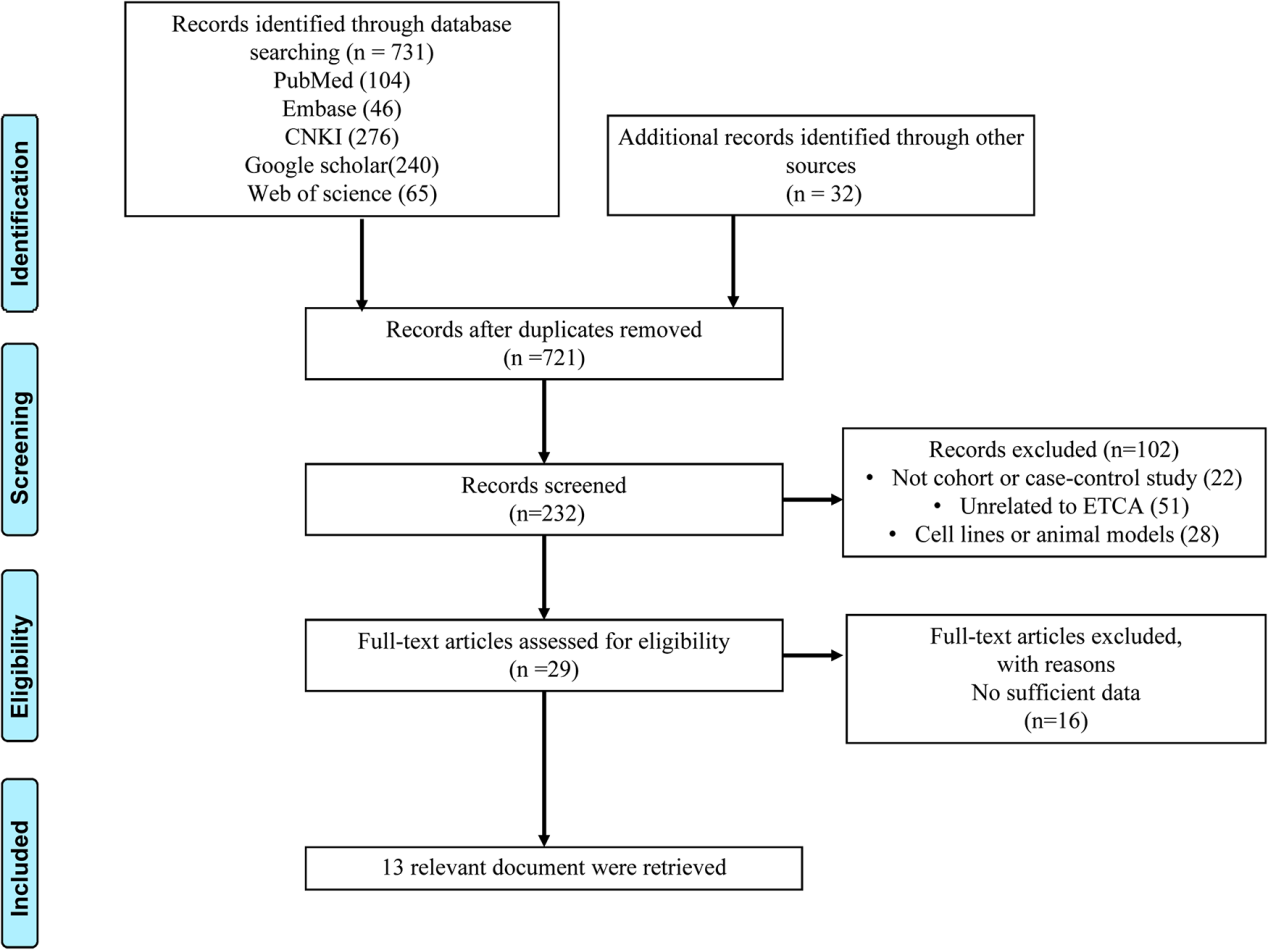
PRISMA flow chart.

The search strategy created 721 relevant articles after removing duplicates. After screening titles and abstracts and excluding duplicate references, 232 articles were identified. We found that some of those studies were relevant to benign disease or other surgical approach. Then, a full-text review was conducted to exclude those that did not meet inclusion criteria, and 13 studies (9, 15–26) were identified. A flow chart for selection and exclusion of studies was shown in Figure 1. Combined with our data, 14 studies comprised of 2,153 patients were finally enrolled for this analysis: 955 in the ETCA group and 1,198 patients in the COT group. The quality assessments and general characteristics of the studies included in the meta-analysis were shown in Table 2.

**Table 2:** Characteristics of the included studies in the meta-analysis.

First author	Year	Country	Group	No. of patients	Age (years), mean ± SD	Operative time mean ± SD	Intraoperative bleeding volume mean ± SD	Number of removed lymph nodes mean ± SD	Postoperative drainage volume mean ± SD	Length of postoperative hospital stay mean ± SD	Hoarseness	Hypocalcemia	Hematoma	Infection	Study design	NOS Score
Zhao et.al	2016	China	ETCA	52	NA	50.1 ± 11.7	13.5 ± 6.3	NA	41.4 ± 7.8	NA	15/200	NA	NA	NA	Retro	6
			COT	60	NA	46.6 ± 13.1	20.1 ± 6.2	NA	55.2 ± 9.2	NA	12/200	NA	NA	NA		
Peng et.al	2018	China	ETCA	56	36.3 ± 7.6	132.1 ± 44.8	NA	5.9 ± 3.5	NA	NA	2/56	8/56	NA	1/56	Retro	7
			COT	56	38.6 ± 8.1	96.7 ± 36.4	NA	7 ± 5.6	NA	NA	1/56	13/56	NA	0/56		
Zhang et.al	2018	China	ETCA	200	31.46 ± 7.75	84.8 ± 19.4	8.72 ± 3.44	NA	62.22 ± 20.1	3.47 ± 0.51	NA	5/200	NA	NA	Retro	8
			COT	200	37.28 ± 6.22	43.8 ± 9.29	6.56 ± 3.24	NA	48.28 ± 13.9	3.17 ± 0.73	NA	7/200	NA	NA		
Li et.al	2019	China	ETCA	44	29.61 ± 6.05	96.27 ± 15.82	26.48 ± 17.47	4.14 ± 1.19	NA	4.66 ± 0.96	NA	NA	NA	NA	Retro	8
			COT	44	32.07 ± 7.75	89.93 ± 16.67	31.3 ± 16.74	4.48 ± 1.18	NA	4.48 ± 0.69	NA	NA	NA	NA		
Xu et.al	2019	China	ETCA	48	36.25 ± 7.37	93.63 ± 20.16	31.04 ± 8.23	7.88 ± 1.72	79.73 ± 18.73	5.3 5 ± 0.76	1/48	3/48	2/48	0/48	Retro	7
			COT	87	47.23 ± 1.09	79.36 ± 15.62	29.47 ± 7.37	7.64 ± 2.44	59.85 ± 20.34	5.36 ± 0.76	4/87	9/87	2/87	1/87		
Gong et.al	2019	China	ETCA	72	36.4 ± 12.7	108.9 ± 12.9	18.3 ± 6.9	5.2 ± 1.6	80.6 ± 18.5	3.8 ± 1.2	1/72	4/72	3/72	0/72	Retro	8
			COT	45	36.2 ± 11.6	75.8 ± 19.2	35.6 ± 3.2	5.8 ± 1.9	85.2 ± 22.4	4 ± 0.9	2/45	3/45	2/45	0/45		
Sun et.al	2019	China	ETCA	119	34.59 ± 7.69	154.32 ± 39.85	NA	6.34 ± 4.1	169.48 ± 55.67	4.27 ± 0.95	0/119	37/119	NA	1/119	Retro	9
			COT	289	45.18 ± 11.5	67.85 ± 21.88	NA	5.83 ± 3.71	117.44 ± 46.18	4.18 ± 1.02	3/289	91/289	NA	0/289		
Chen et.al	2019	China	ETCA	28	38.2 ± 5.1	115.7 ± 32.4	28.4 ± 6.2	5.4 ± 1.5	93.7 ± 21.2	NA	2/28	1/28	2/28	0/28	Retro	7
			COT	35	40.4 ± 6.8	95.3 ± 27.6	33.1 ± 7.6	5.2 ± 1.4	54.8 ± 17.5	NA	2/35	2/35	1/35	3/35		
Xia et.al	2019	China	ETCA	40	45.54 ± 4.62	145.42 ± 12.32	61.54 ± 5.8	5.2 ± 0.3	32.43 ± 2.54	NA	1/40	2/40	1/40	1/40	Retro	7
			COT	40	46.63 ± 5.01	98.53 ± 7.63	87.33 ± 7.24	5.1 ± 0.3	49.64 ± 4.13	NA	3/40	4/40	2/40	4/40		
Luo et.al	2020	China	ETCA	54	NA	NA	27.44 ± 5.21	3.98 ± 1.46	NA	NA	1/54	NA	1/54	0/54	Retro	6
			COT	54	NA	NA	45.39 ± 5.18	5 ± 1.28	NA	NA	4/54	NA	2/54	1/54		
Yuan et.al	2020	China	ETCA	72	31.86 ± 0.09	137.8 ± 20.55	52.51 ± 20.73	4.52 ± 0.75	28.72 ± 6.57	4.14 ± 0.42	5/72	4/72	0/72	1/72	Retro	8
			COT	128	34.66 ± 9.76	108.14 ± 10.84	60.47 ± 18.96	4.67 ± 0.59	38.97 ± 8.78	4.3 ± 0.81	9/128	8/128	1/128	2/128		
Liu et.al	2020	China	ETCA	30	32.0 ± 7.9	162 ± 36.9	15.9 ± 8.2	8.5 ± 5.7	178.1 ± 50.4	4.4 ± 0.7	0/30	2/30	5/30	NA	Retro	9
			COT	30	42.3 ± 8.6	79.4 ± 28.6	13.7 ± 7.5	7.5 ± 6.5	127 ± 30.1	4.1 ± 0.9	1/30	2/30	0/30	NA		
Qu et.al	2021	China	ETCA	67	44.34 ± 9.82	115.2 ± 21.3	28.2 ± 6.9	5.4 ± 1.6	53.8 ± 24.1	NA	2/67	8/67	1/67	1/67	Retro	8
			COT	67	45.56 ± 8.91	95.6 ± 17.4	34.3 ± 7.9	5.1 ± 1.3	91.6 ± 29.3	NA	2/67	13/67	3/67	3/67		
Yuan et al	2022	China	ETCA	73	32.51 ± 6.03	114.72 ± 25.62	18.38 ± 7.77	8.84 ± 6.52	195.75 ± 80.1	3.68 ± 0.88	2/73	NA	NA	1/73	Retro	9
			COT	63	36.00 ± 8.01	102.96 ± 22.79	35.67 ± 11.42	6.41 ± 5.47	137.83 ± 52.09	3.59 ± 1.10	2/63	NA	NA	1/63		

ETCA, endoscopic thyroidectomy by complete areola approach; COT, conventional open thyroidectomy; retro, retrospective; NOS score, Newcastle–Ottawa scale (NOS) score; NA, not available

### Outcomes of meta-analysis

**Figure 2:**
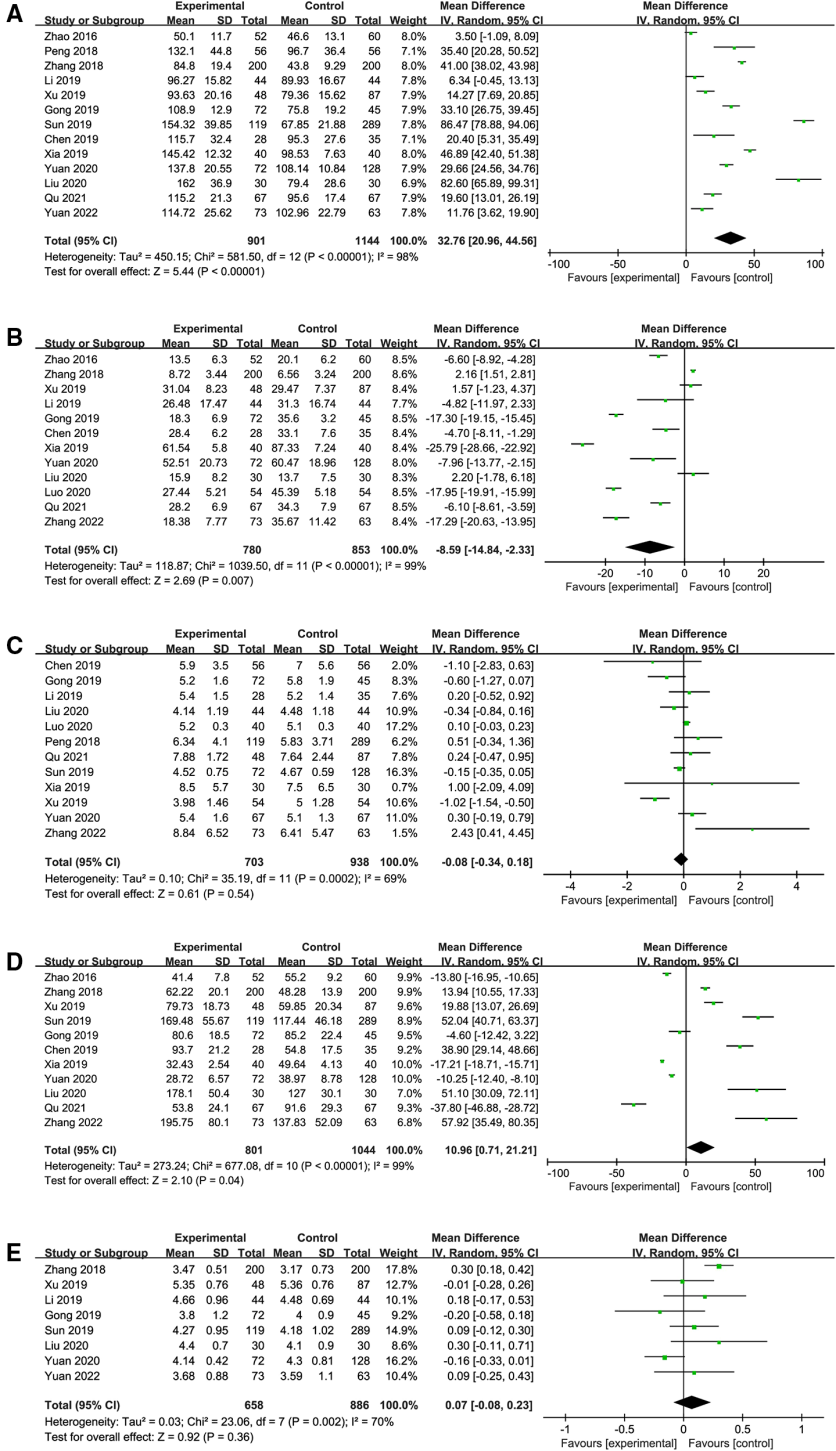
Forest plot of the meta-analysis. Experimental group: ETCA group; Control group: COT group. (**A**) operative time; (**B**) intraoperative bleeding volume; (**C**) removed lymph node; (**D**) postoperative drainage volume; (**E**) length of postoperative hospital stay.

**Figure 3:**
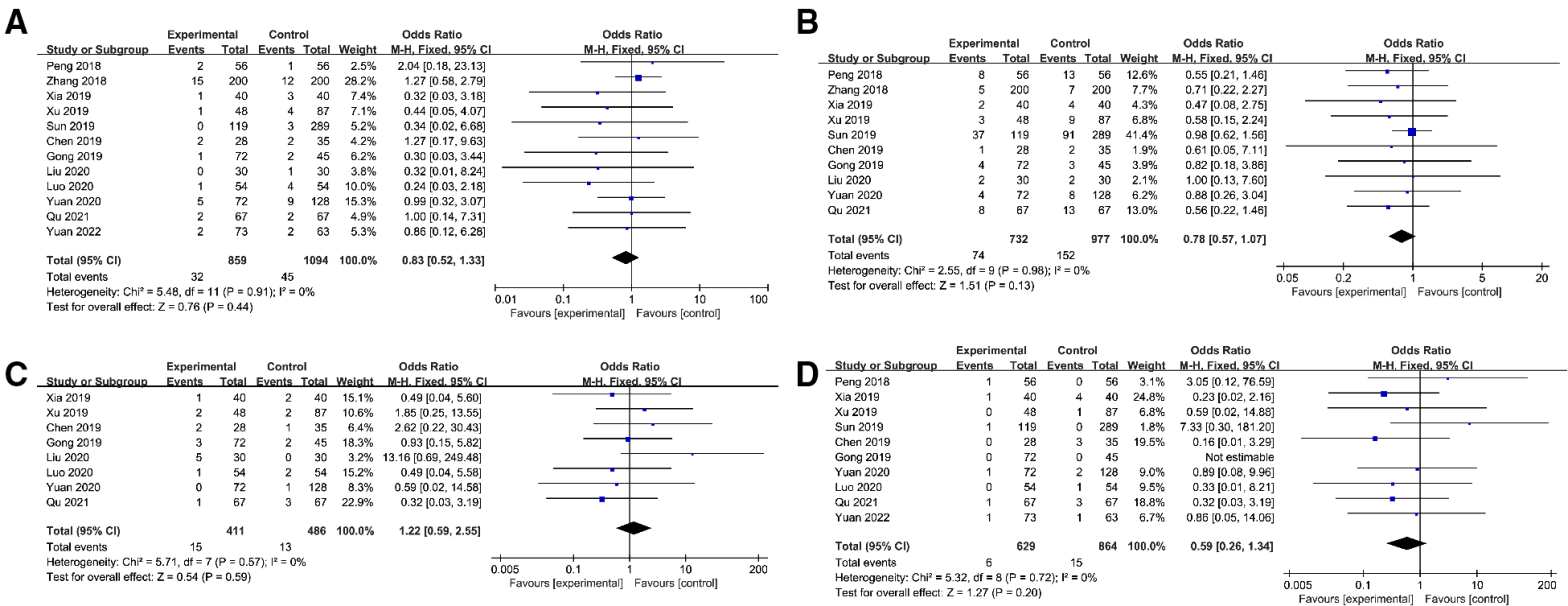
Forest plot of the meta-analysis. Experimental group: ETCA group; Control group: COT group. (**A**) hoarseness; (**B**) hypocalcemia; (**C**) hematoma; (**D**) infection.

The result of the meta-analysis in thirteen studies (9, 15–24, 26) indicated that the operative time in the ETCA group was significantly longer than that in the COT group (WMD:32.76, 95% CI: 20.96–44.56, *P *< 0.00001, *I^2 ^*= 98%) (Figure 2A). Based on the overall result of twelve studies, the intraoperative bleeding volume in the ETCA group was higher than in the COT group (15, 17–26) (WMD: −8.59 95% CI: −14.84–−2.33 *P *= 0.007, *I^2 ^*= 99%) (Figure 2B). Twelve studies (9, 16, 19–26) assessed the number of removed lymph nodes and the pooled data showed the number of removed lymph nodes in ETCA group is comparable to COT groups (WMD: −0.08, 95% CI: −0.34–0.18, *P *= 0.54 *I^2 ^*= 69%.) (Figure 2C). Eleven studies (9, 15, 17, 19–24, 26) presented the postoperative drainage volume and revealed a smaller drainage volume in the COT group (WMD:10.96, 95% CI: 0.71–21.21, *P *= 0.04 *I^2 ^*= 99%) (Figure 2D). Eight studies (9, 17–20, 23, 24) described the length of postoperative hospital stay, and the combined results of these studies showed that there was no significant difference between ETCA group and COT group. (WMD: 0.07, 95% CI: −0.08–0.23, *P *= 0.36, *I*^2 ^= 70%) (Figure 2E). Incidences of several surgical complications, including hoarseness, hypocalcemia, hematoma, and infection, were calculated in this meta-analysis. Twelve studies (9, 16, 17, 19–26) reported the rate of hoarseness and the pooled data showed there was no significant difference between the two groups in the rate of hoarseness (OR 0.83, 95% CI: 0.52–1.33, *P *= 0.44, *I^2 ^*= 0%) (Figure 3A). Ten studies (9, 16, 17, 19–24, 26) reported the postoperative hypocalcemia rate, which found no significant differences between the two groups (OR: 0.78, 95% CI: 0.57–1.07, *P *= 0.13, *I^2 ^*= 0%) (Figure 3B). Eight studies (19–26) calculated the rate of hematoma, and there was no difference between the two groups (OR: 1.22, 95% CI: 0.59–2.55, *P *= 0.59, *I^2 ^*= 0%.) (Figure 3C). Ten studies (9, 16, 19–22, 24–26) presented the rate of postoperative infection, showing comparable rates between the two groups (OR: 0.59, 95% CI: 0.26–1.34, *P *= 0.20, *I^2 ^*= 0%) (Figure 3D). Details about the result of meta-analysis showed in Table 3.

**Table 3:** the results of the meta-analysis between the two groups.

Outcomes	No. of studies	No. of patients	WMD/OR	95%CI	*P* Value	*I*^2^ (%)
Operative time	13	2045	32.76	20.96–44.56	<0.00001	98%
Intraoperative bleeding volume	12	1633	−8.59	−14.84–2.33	0.007	99%
Number of removed lymph nodes	12	1641	−0.08	−0.34–0.18	0.54	69%
Postoperative drainage volume	11	1845	10.96	0.71–21.21	0.04	99%
Length of postoperative hospital stay	8	1544	0.07	−0.08–0.23	0.36	70%
Hoarseness	12	1895	0.83	0.52–1.33	0.44	0%
Hypocalcemia	10	1651	0.78	0.57–1.07	0.13	0%
Hematoma	8	839	1.22	0.59–2.55	0.59	0%
Infection	10	1465	0.59	0.26–1.34	0.20	0%

WMD, weighted mean difference; CI, confidence interval; OR, Odds Ratio.

### Sensitivity analyses and publication bias

**Figure 4:**
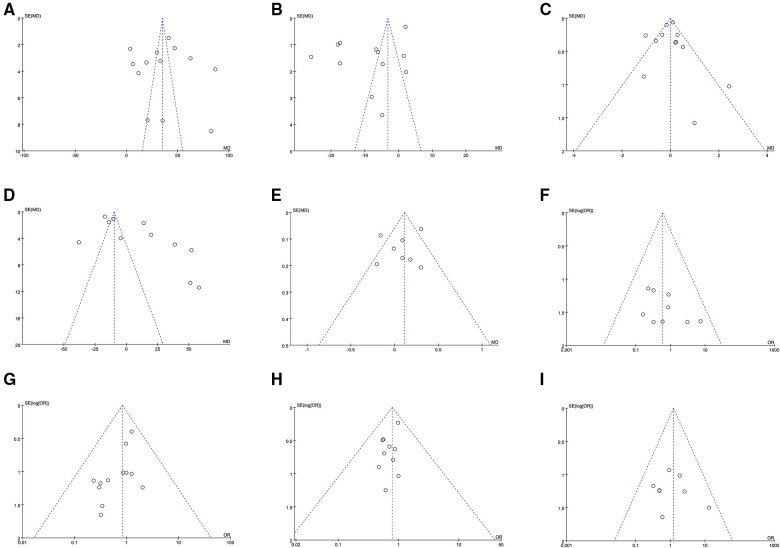
Funnel plot of all outcomes in all included studies. OR: odds ratio. (**A**) operative time; (**B**) intraoperative bleeding volume; (**C**) removed lymph node; (**D**) postoperative drainage volume; (**E**) length of postoperative hospital stay. (**F**) hoarseness; (**G**) hypocalcemia; (**H**) hematoma; (**I**) infection.

A sensitivity analysis was conducted by deleting individual studies and the replacement of effect models, whereas the overall statistical significance did not change, indicating that the results were robust and reliable. The funnel plot of the studies based on the hoarseness did not find any obvious publication bias (Figure 4).

## Discussion

There are several types of endoscopic thyroidectomies that can be applied to avoid scarring on the neck (27–29). Among those approaches, ETCA has been widely used because of its advantages, including concealed incisions and lower risk of scar hyperplasia due to less skin tension in the areola. A growing number of publications on safety and treatment outcomes of ETCA have been published in recent years. In this study, we performed a systematic review and meta-analysis of the studies comparing treatment outcomes for ETCA and COT published until June 2022. Additionally, we also enrolled clinical data from Chongqing General Hospital in this meta-analysis to pursue a more credible conclusion.

The results of this meta-analysis showed that the operative time was significantly longer in the ETCA group than that in the COT group, which is consistent with previous similar analyses (30–32). Jiang et al. had concluded a similar result and reported that creating the flap for ET, a procedure not required for conventional thyroidectomy, needs an additional time (33). Furthermore, meticulous bleeding control and accurate lymph-node dissection require longer operation time (34). The instruments of ETCA were challenging to manipulate, prolonging the operation time because of the narrow artificially created operating space. However, the operative time of ETCA could decrease dramatically for upgrading experience and advances in instrument technology. The intraoperative blood loss of the ETCA group was less than that of the COT group in this meta-analysis. The outcome of our clinical data and a retrospective analysis of 134 cases of PTC reported by Qu et al. both revealed the similar conclusion (26). The possible reason includes following aspects. First, the magnification effect of endoscopic surgery allows small vessels to be clearly visualized. Second, the ultrasonic scalpel has good hemostatic ability for glands and microvasculature (9).

The results of the meta-analysis revealed that the postoperative drainage volumes in the ETCA group was more than that of the COT group which is similar to the outcome of our data. The possible reason is that a large amount of sterile water is usually used to flush the wound to locate the bleeding site at the end of the ETCA procedure. Then, some of the sterile water would remain in the tissue space under the pressure of carbon dioxide, thereby gradually drained out after the operation.

Complete dissection of metastatic lymph nodes is vital for the treatment of DTC because it influences the prognosis of patients and the recurrence of the tumor (35). Meanwhile, ETCA and its effectiveness of central lymph node dissection remains controversial. Some studies have concluded that the number of the dissected central lymph nodes in endoscopic surgery is less than that in conventional open surgery and believed that the surgical view of complete areola approach was limited due to the obstruction of the sternum, so the central lymph nodes were not able to be completely removed (33, 36, 37). However, this meta-analysis revealed that the number of lymph nodes removed in the ETCA group was comparable to the COT group, similar to the findings in the previous studies (9, 34). A retrospective study of 119 ETCA and 289 COT patients reported by Sun et al. has shown the same result and revealed that a standard 10-mm 30° laparoscope could provide excellent visibility so that the lymph nodes could be clearly visualized and dissected (9). Incredibly, the number of lymph nodes dissected in ETCA was even more than that in COT according to the result of our clinical data. The magnification effect of the endoscope allows the laryngeal nerve and adipose tissue to be clearly visualized, which might lead to a propensity for the surgeons to remove more lymph nodes. More extensive data from more high-quality multi-center randomized controlled studies are needed to confirm the feasibility of ETCA on central lymph node dissection.

The safety of the RLN by endoscopic surgery remains contentious. The study reported by Li et al. showed that ET shared a higher incidence of transient RLN palsy than COT due to the thermal damage caused by the ultrasonic scalpel (38). Since the surgical field of the complete areola approach is unfamiliar for the surgeon compared to COT, it might affect the judgment of the anatomical location of the RLN and increase the chance of injury to the RLN. Additionally, the central lymph nodes were pulled laterally from the trachea during ETCA, thereby increasing the risk of traction injury to the RLN (37). Despite this expectation, according to the result of this meta-analysis, there was no significant difference in the incident rates of transient recurrent nerve palsy between the ETCA group and the COT group. This conclusion was also shared by some previous studies (9, 30, 31, 34, 39). As surgeon gains experience, one can maintain the integrity of RLN with the help of the nerve monitor, achieving the same results as COT.

There are several limitations in this study. First, all included studies were non-randomized controlled trials, and all included studies were performed in China, potentially limiting the clinical outcomes to patients of Chinese descent and may not apply to other ethnic groups. Second, differences in surgeon experience will influence the outcome of the research. Third, some particular complications of ETCA, such as subcutaneous emphysema, hypercarbia, and tumor seeding, were not analyzed in this study.

## Conclusion

Results of our clinical data and meta-analysis both conclude that the ETCA is not as good as the COT in terms of operative time and postoperative drainage volume. However, ETCA is comparable to COT in terms of the length of postoperative hospital stay, number of removed lymph nodes and incidence of surgical complications. In addition, ETCA reduces surgical bleeding and provides an excellent cosmetic appearance, which is a unique advantage over COT. The above results suggest that ETCA is an effective and safety alternative for patients with DTC compared with COT. Larger size and long-term randomized clinical trials are needed to prove the clinical value of the ETCA in the treatment of DTC in the future.

## Data Availability

The original contributions presented in the study are included in the article, further inquiries can be directed to the corresponding author/s.
